# African swine fever virus I267L acts as an important virulence factor by inhibiting RNA polymerase III-RIG-I-mediated innate immunity

**DOI:** 10.1371/journal.ppat.1010270

**Published:** 2022-01-28

**Authors:** Yong Ran, Dan Li, Mei-Guang Xiong, Hua-Nan Liu, Tao Feng, Zheng-Wang Shi, Yu-Hui Li, Huang-Ning Wu, Su-Yun Wang, Hai-Xue Zheng, Yan-Yi Wang

**Affiliations:** 1 Key Laboratory of Special Pathogens and Biosafety, Wuhan Institute of Virology, Chinese Academy of Sciences, Wuhan, China; 2 African Swine Fever Regional Laboratory of China, Wuhan Institute of Virology, Chinese Academy of Sciences, Wuhan, China; 3 State Key Laboratory of Veterinary Etiological Biology, OIE/National Foot and Mouth Disease Reference Laboratory, Lanzhou Veterinary Research Institute, Chinese Academy of Agricultural Sciences, Lanzhou, China; 4 African Swine Fever Regional Laboratory of China, Lanzhou Veterinary Research Institute, Chinese Academy of Agricultural Sciences, Lanzhou, China; 5 University of Chinese Academy of Sciences Beijing, China; Kansas State University, UNITED STATES

## Abstract

ASFV is a large DNA virus that is highly pathogenic in domestic pigs. How this virus is sensed by the innate immune system as well as why it is so virulent remains enigmatic. In this study, we show that the ASFV genome contains AT-rich regions that are recognized by the DNA-directed RNA polymerase III (Pol-III), leading to viral RNA sensor RIG-I-mediated innate immune responses. We further show that ASFV protein I267L inhibits RNA Pol-III-RIG-I-mediated innate antiviral responses. I267L interacts with the E3 ubiquitin ligase Riplet, disrupts Riplet-RIG-I interaction and impairs Riplet-mediated K63-polyubiquitination and activation of RIG-I. I267L-deficient ASFV induces higher levels of interferon-β, and displays compromised replication both in primary macrophages and pigs compared with wild-type ASFV. Furthermore, I267L-deficiency attenuates the virulence and pathogenesis of ASFV in pigs. These findings suggest that ASFV I267L is an important virulence factor by impairing innate immune responses mediated by the RNA Pol-III-RIG-I axis.

## Author summary

African swine fever virus (ASFV) is a large DNA virus that is highly contagious and pathogenic in domestic pigs with a lethality rate up to 100%. Infection of ASFV has become a global threat with devastating economic and ecological consequences. Unfortunately, commercially available, safe and efficacious vaccines are still lacking so far. How this virus is sensed by the host innate immune system as well as why this virus is so virulent remains enigmatic. Understanding some basic aspects of ASFV-host interaction is helpful for vaccine development. In this study, we found that the highly AT-enriched ASFV genomic DNA is sensed by DNA-directed RNA polymerase III (Pol-III) that transcribes the AT-rich genomic DNA into RNA, which is then recognized by the pattern recognition receptor RIG-I, leading to innate immune responses. This represents one of few examples whereby a DNA virus is primarily sensed by the Pol-III-RIG-I axis. ASFV early gene-encoded protein I267L antagonizes RIG-I-mediated innate immune responses. I267L interacts with Riplet, an E3 ligase essential for RIG-I activation. This disrupts the interaction of Riplet with RIG-I, and impairs Riplet-mediated K63-linked polyubiquitination and activation of RIG-I. Consistently, I267L-deficient ASFV induces higher levels of IFN-β and displays compromised replication both in primary porcine alveolar macrophages (PAMs) and pigs comparing with wild-type ASFV. Furthermore, I267L-deficiency attenuates the virulence and pathogenesis of ASFV in pigs. These results reveal a critical mechanism responsible for the virulence of ASFV, and suggest that deletion of I267L may serve as a strategy to develop attenuated vaccines for ASFV.

## Introduction

African swine fever virus (ASFV), the only member of the *Asfarviridae* family, is a highly pathogenic large DNA virus that causes contagious African swine fever (ASF) in domestic pigs with a lethality rate up to 100%. Due to the absence of effective vaccines for ASFV, ASF has been spreading in Europe and Asia in recent years, becoming a global threat with devastating economic and ecological consequences [1–3].

ASFV is a large, enveloped virus with icosahedral morphology and an average diameter of 200 nm. The ASFV genome consists of a linear, non-segmented, double-stranded (ds) DNA with length ranging from 170 to 193 kilobase pairs which encodes more than 150 proteins, including enzymes and factors required for genome replication and transcription, and proteins that have various roles in manipulation of the immune responses [1–3].

ASFV has tropism for cells of the myeloid lineage, especially macrophages and monocytes [4]. It has been well known that infection of these cells by various viruses induces large amounts of type I interferons (IFNs) and inflammatory cytokines [5]. However, ASFV infection in pigs induces low levels of type I IFNs, which may contribute to its virulence [6]. It remains unknown as to whether and how ASFV is sensed by cellular pattern recognition receptors (PRRs) and whether the virulence of the virus is due to evasion of the innate immune system. Previously, it has been shown that viral DNA can be sensed by different PRRs located at particular cellular compartments [7,8]. For examples, TLR9 detects unmethylated DNA in the endolysosomes [9]; cyclic GMP-AMP synthase (cGAS) senses cytosolic DNA in a sequence independent manner [10]; DNA-dependent RNA polymerase III can transcribe AT-rich DNA template to produce 5’-ppp-RNA, which serves as an agonist for the RNA sensor RIG-I [11]. In addition to viral nucleic acids, infection of certain viruses can trigger mitostress, resulting in release of mitochondrial DNA that is sensed by cGAS and the subsequent innate antiviral response [12]. Whether these mechanisms contribute to innate immune responses against ASFV is unclear.

Previous studies have demonstrated that multiple ASFV-encoded proteins can antagonize host innate immune responses by inhibiting production of type I IFNs as well as inflammatory cytokines. It has been reported that ASFV MGF360 and MGF530/505 inhibit both type I IFNs and their downstream antiviral interferon stimulated genes (ISGs) [13]; ASFV DP96R targets both TBK1 and IKKβ to negatively regulate induction of antiviral cytokines [14]; and ASFV A238L inhibits transcriptional factors NF-κB, c-JUN, and NFAT [15,16]. However, whether ASFV targets the upstream viral DNA sensing components for immune evasion is unknown.

In this study, we found that the ASFV genome is enriched with AT-rich sequences, which could be recognized by host DNA-directed RNA Pol-III, leading to RIG-I-mediated innate immune responses. We also identified ASFV-encoded protein I267L as a potent inhibitor of RNA Pol-III-RIG-I signaling. I267L interacted with the E3 ubiquitin ligase Riplet, disrupted its interaction with RIG-I, and thus impaired Riplet-mediated K63-linked polyubiquitination and activation of RIG-I. Infection of pigs with I267L-deficient ASFV showed increased serum IFN-β level, viral replication and attenuated virulence and pathogenesis. Our findings suggest that the RNA Pol-III-RIG-I axis mediates innate immune sensing of the ASFV genome whereas evasion of this sensing by I267L contributes to virulence and pathogenesis of ASFV.

## Results

### AT-rich regions of the ASFV genome activates RNA Pol-III-RIG-I-mediated innate antiviral responses

**Fig 1:**
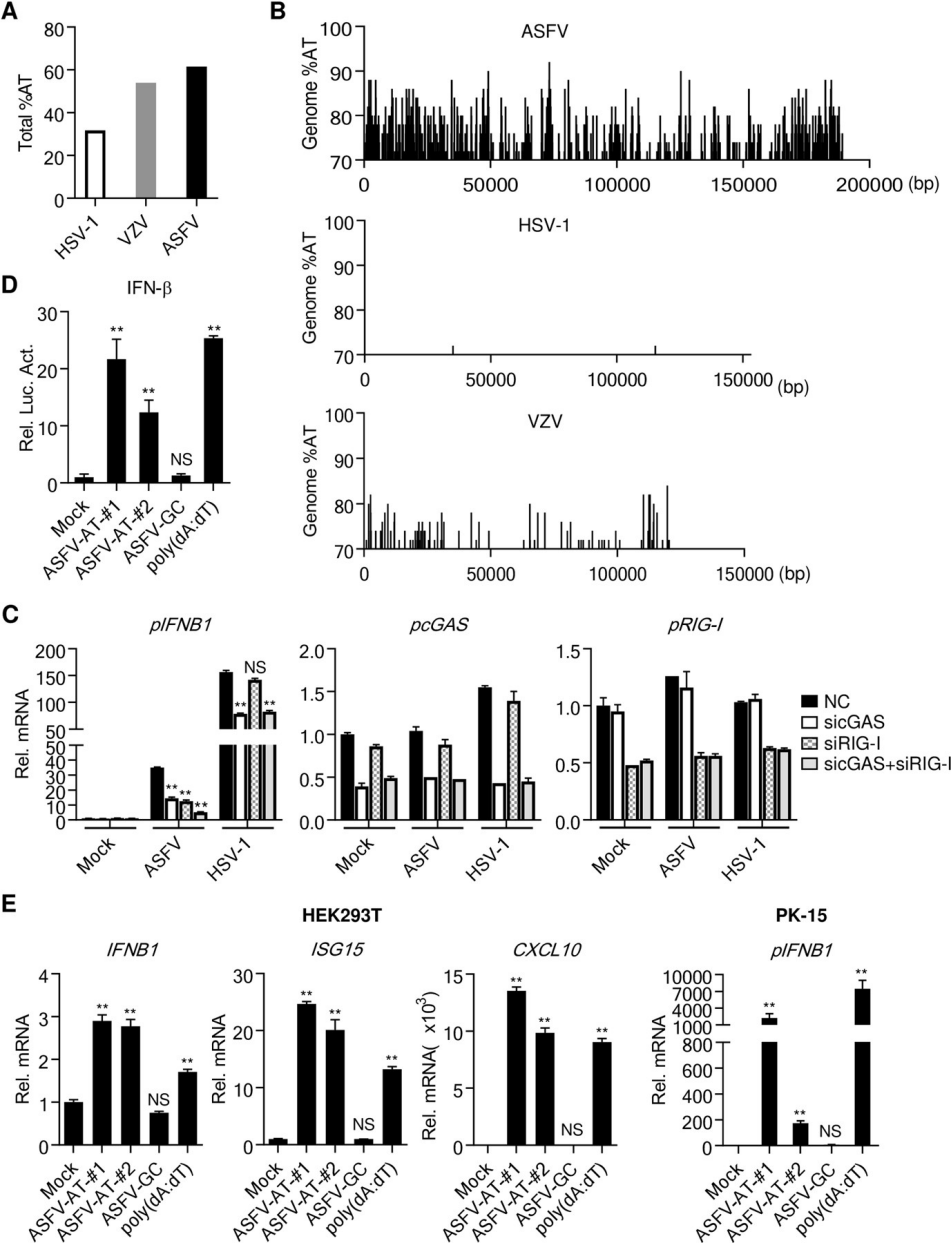
AT-rich regions of the ASFV genome induce IFN-β. (**A**) Comparison of the total AT-content of the ASFV genome with genomes of HSV-1, EBV and VZV. (**B**) The content and distribution of AT-rich regions in the genomes of ASFV were compared with those in the genomes of HSV-1 and VZV using the EMBOSS Isochore algorithm. Regions with AT content more than 70% are shown. (**C**) Effects of knockdown of cGAS or RIG-I on *IFNB1* transcription induced by ASFV or HSV-1 infection. PAMs (1×10^6^) were transfected with the indicated siRNA (final concentration, 25 nM each) for 48 hours, and then the cells were infected with ASFV or HSV-1 (MOI = 0.1) for 4 hours. The mRNA levels of *IFNB1*, *cGAS*, and *RIG-I* were analyzed by qPCR. (**D**) The AT-rich DNA of the ASFV genome activates the IFN-β promoter. HEK293T (1×10^5^) were transfected with the IFN-β promoter luciferase plasmid. Twenty-four hours later, these cells were stimulated with transfected dsDNA as indicated (1 μg/ml) for 10 hours before luciferase assays were performed. ASFV-AT and ASFV-GC are short for ASFV/DNA-AT and ASFV/DNA-GC respectively. (**E**) qPCR analysis of mRNA levels of the indicated genes stimulated by transfected dsDNA (1 μg/ml) for 6 hours in HEK293T and PK-15 cells.

A recent report has shown that the attenuated ASFV strain (NH/P68) but not the virulent strain (Armenia/07) induces innate antiviral responses and this is inhibited by an inhibitor of cGAS. They therefore conclude that ASFV activates cGAS-mediated innate immune responses which are evaded by virulent ASFV [17]. However, this study did not provide evidence on the direct involvement of cGAS in sensing ASFV genomic DNA, and whether ASFV is monitored by other innate immune sensors is unknown. Sequence analysis indicated that the overall AT content of the ASFV genome is up to 61.6%, which is markedly higher than that of varicella zoster virus (VZV) (54.0%) and herpes simplex virus type 1 (HSV-1) (31.7%) (Fig 1A). It has been previously demonstrated that the AT-rich islands of VZV genome can be recognized by RNA Pol-III [18]. Interestingly, the ASFV genome contains numerous AT-rich islands with AT content between ~70–90%, and both the frequency of AT-rich islands and the ratios of AT content are markedly higher than that of VZV (Fig 1B). In contrast, AT-rich islands are barely found in the genome of HSV-1 (Fig 1B). These analyses suggest that the ASFV genome may be sensed by the RNA Pol-III-RIG-I axis. To evaluate the contributions of cGAS and RNA Pol-III-RIG-I to sensing of ASFV infection, we examined the effects of knockdown of cGAS or RIG-I on ASFV-induced transcription of the *IFNB1* gene. The results indicated that knockdown of either cGAS or RIG-I inhibited ASFV-induced transcription of the *IFNB1* gene to similar levels, and simultaneous knockdown of both had collaborative inhibitory effects (Fig 1C). By contrast, HSV-1-induced transcription of the *IFNB1* gene was impaired by knockdown of cGAS but not RIG-I (Fig 1C). These results suggest that ASFV is sensed by both cGAS and RNA Pol-III-RIG-I pathways.

To investigate whether ASFV genomic DNA is sensed by the RNA Pol-III-RIG-I axis, we synthesized a GC-rich (61 bp) and two AT-rich (85 and 113 bp respectively) dsDNA fragments of the ASFV genome to examine their abilities to stimulate innate immune responses. Reporter assays indicated that transfection of the synthesized AT-rich DNA (ASFV/DNA-AT) and poly(dA:dT) but not GC-rich DNA (ASFV/DNA-GC) activated the IFN-β promoter in HEK293T cells which contain intact RNA Pol-III-RIG-I pathways but lack cGAS and MITA (also called STING), an essential adaptor in cGAS-triggered innate immune responses [7,8,10,11,19–21] (Fig 1D). Consistently, transfection of ASFV/DNA-AT and poly(dA:dT) but not ASFV/DNA-GC induced transcription of *IFNB1*, *ISG15* and *CXCL10* genes in HEK293T cells (Fig 1E). Similarly, ASFV/DNA-AT and poly(dA:dT) but not ASFV/DNA-GC induced transcription of *IFNB1* gene in porcine PK-15 cells (Fig 1E). These results suggest that AT-rich but not GC-rich ASFV DNA can induce innate immune responses in a cGAS-independent manner in these cells.

**Fig 2:**
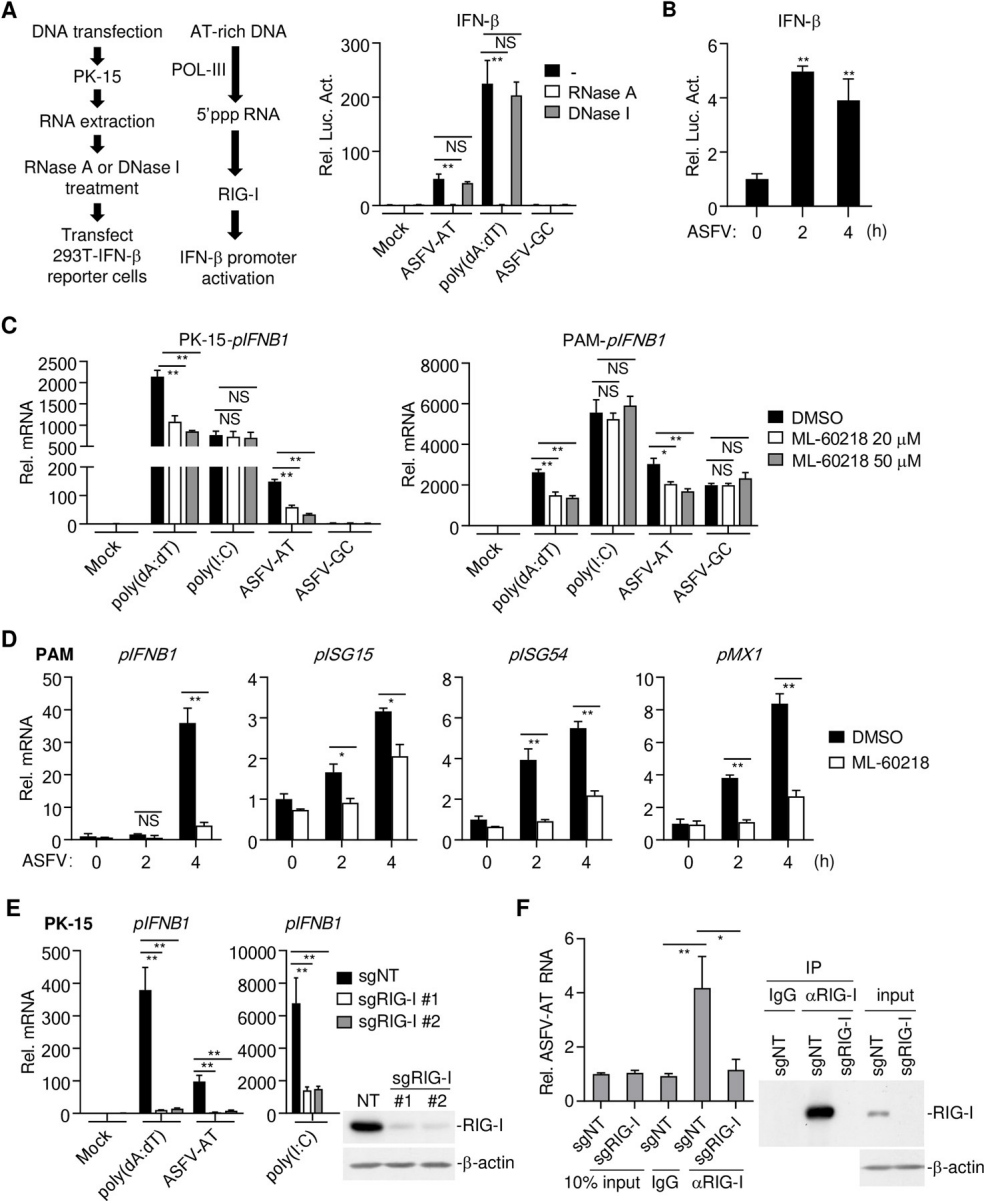
AT-rich regions of the ASFV genome activate RNA Pol-III-RIG-I axis. (**A**) RNAs extracted from poly(dA:dT)-, ASFV-AT-, ASFV-GC (1 μg/ml) or mock-transfected PK-15 cells (2×10^6^) were treated with RNase A or DNase I at 37°C for 1 hour. The RNAs were then transfected into HEK293T (2 μg/ml) that transfected with IFN-β-luciferase reporter. Six-hours later, luciferase assays were performed. The flow diagram of experiment was shown on the left. ASFV-AT indicates ASFV-AT-#1 hereafter. (**B**) PAMs (2×10^6^) were mock infected or infected with ASFV (MOI = 0.1) for the indicated times. RNAs were extracted and treated with DNase (200 U/ml/mg RNA) at 37°C for 1 hour. The RNAs were then transfected into HEK293T (2 μg/ml) that transfected with IFN-β-luciferase reporter. Six-hours later, luciferase assays were performed. (**C**) PK-15 cells or PAMs (1×10^6^) were pre-treated with the indicated concentrations of the RNA Pol-III inhibitor ML-60218 for 6 hours before transfection with poly(dA:dT), ASFV-AT, ASFV-GC or poly(I:C) (1 μg/ml) for 4 hours. The mRNA levels of *IFNB1* were analyzed by RT-qPCR. (**D**) PAMs (2×10^6^) were pre-treated with ML-60218 (50 μM) for 6 hours before infection with ASFV (MOI = 0.1) for the indicated times. The mRNA levels of *IFNB1*, *ISG15*, *ISG54*, and *MX1* were analyzed by RT-qPCR. (**E**) The effects of RIG-I-deficiency on poly(dA:dT)-, ASFV-AT- or poly(I:C)-stimulated IFN-β induction. Two sgRNA were used to knockdown RIG-I in PK-15 cells using CRISPR/Cas9 method (sgNT, non-targeting sgRNA). The mRNA levels of *IFNB1* were analyzed by RT-qPCR after the cells were stimulated with transfected DNA or RNA analogs poly(I:C) for 6 hours. The knockdown efficiency of RIG-I was confirmed by western blot. (**F**) PK-15 cells (2×10^7^) prepared as in (E) were transfected with ASFV-AT-#2 for 3 hours. RIP experiments were performed using the RIG-I antibody, and specific primers were used to detect the enrichment of ASFV-AT by RIG-I in cells. The expression of RIG-I in lysates and immunoprecipitations was detected by western blot analysis.

We next transfected PK-15 cells with poly(dA:dT), ASFV/DNA-AT or ASFV/DNA-GC, and isolated the RNAs from these transfected cells. The RNAs were then treated with RNase A or DNase I or left untreated before they were transfected into HEK293T cells to examine their abilities to activate the IFN-β promoter by reporter assays. The results indicated that RNA from poly(dA:dT)-, ASFV/DNA-AT- but not ASFV/DNA-GC-transfected cells activated the IFN-β promoter, which was abolished by RNase A but not DNase I treatment of the RNAs before transfection (Fig 2A). Similarly, we isolated RNAs from porcine alveolar macrophages (PAMs) infected with ASFV and examined their abilities to activate the IFN-β promoter by reporter assays. The results indicated that ASFV-infected PAMs produced RNAs that could activate the IFN-β promoter in HEK293T cells (Fig 2B). Additionally, ML-60218, a specific inhibitor of RNA Pol-III, markedly inhibited transcription of *IFNB1* gene induced by transfected poly(dA:dT) and ASFV/DNA-AT but not poly(I:C) or ASFV/DNA-GC in porcine PK-15 cells or PAMs respectively (Fig 2C). Transcription of downstream antiviral genes induced by ASFV infection including *IFNB1*, *ISG15*, *ISG54* and *MX1* was also inhibited by ML-60218 in PAMs (Fig 2D). Knockout of RIG-I in PK-15 cells by the CRISPR/Cas9 method impaired transcription of *IFNB1* gene induced by transfection of poly(dA:dT), ASFV/DNA-AT and poly(I:C) (Fig 2E). In addition, RNA immunoprecipitation (RIP) experiments indicated that RIG-I bound to RNAs transcribed from ASFV AT-rich DNA (Fig 2F). Taken together, these results suggest that the AT-rich ASFV DNA is transcribed to RNA by Pol-III, which is sensed by RIG-I, leading to innate immune responses.

### ASFV I267L targets Riplet to antagonize the RNA Pol-III-RIG-I axis

**Fig 3:**
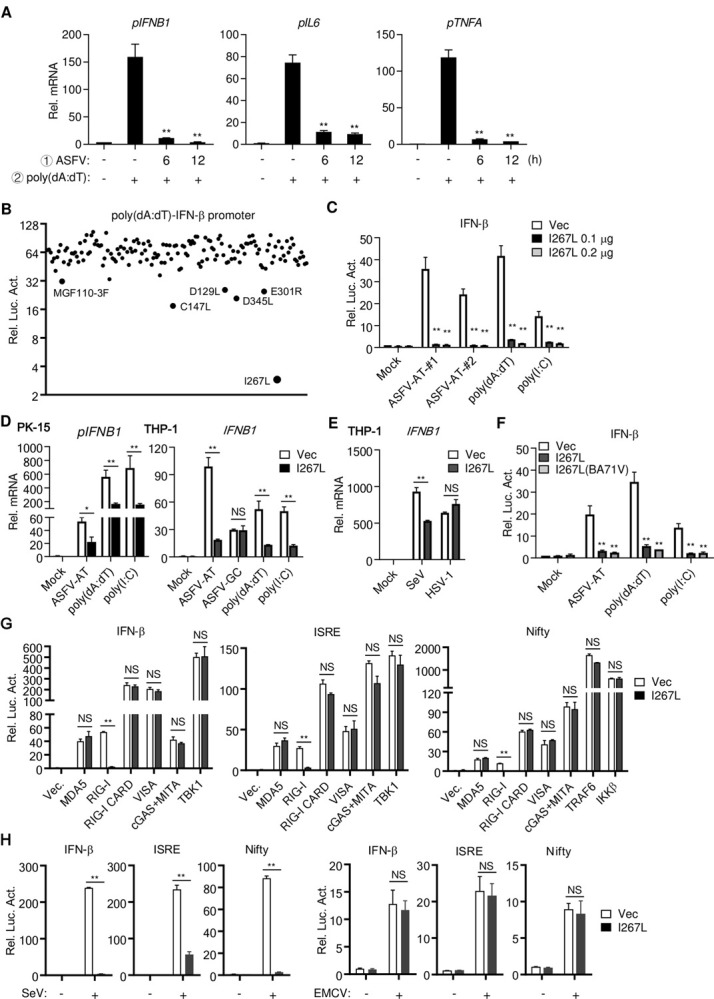
ASFV antagonizes RNA Pol-III-RIG-I axis. (**A**) ASFV infection inhibits poly(dA:dT)-induced transcription of *IFNB1*, *IL6* and *TNFA*. PAMs (2×10^6^) were uninfected or infected with ASFV (MOI = 0.1) for the indicated times, and then were stimulated with transfected poly(dA:dT) (1 μg/ml) for 4 hours before RT-qPCR were performed. (**B**) Screening of ASFV proteins that inhibits poly(dA:dT)-induced activation of the IFN-β promoter. HEK293T cells were transfected with plasmids of individual ASFV cDNA (0.1 μg), the IFN-β luciferase reporter (0.05 μg) and TK reporter (0.01 μg/ml). Twenty-four hours later, cells were left untreated or stimulated with transfected poly(dA:dT) (1 μg/ml) for 10 hours before luciferase assays were performed. The proteins that showed more than two-fold inhibition are labeled in the figure. (**C**) The effects of I267L on activation of the IFN-β promoter stimulated by transfected ASFV-AT, poly(dA:dT), and poly(I:C) (1 μg/ml). The experiments were similarly performed as in (B). (**D**) PK-15 and THP-1 cells (1×10^6^) stably transduced with vector or I267L were stimulated with transfected ASFV-AT, poly(dA:dT), and poly(I:C) (1 μg/ml) for 6 hours before RT-qPCR were performed. (**E**) THP-1(1×10^6^) stably transduced with vector or I267L were infected with SeV or HSV-1 (MOI = 0.1) for 6 hours before qPCR was performed. (**F**) The effects of I267L encoded by ASFV genotype I (Ba71V) and genotype II (CN/GS/2018) on activation of the IFN-β promoter stimulated by transfected ASFV-AT, poly(dA:dT), and poly(I:C) (1 μg/ml). The experiments were similarly performed as in (B). (**G**) HEK293T cells (1×10^5^) were co-transfected with the indicated reporters, expression plasmids and empty vectors or I267L expression plasmids (0.05 μg). Luciferase assays were performed 24 hours after transfection. pNifty-luciferase reporter: a reporter to indicate NF-κB activation, which contains five NF-κB binding sites. (**H**) HEK293T cells (1×10^5^) were co-transfected with the indicated reporters, empty vectors or I267L expression plasmids (0.05 μg). Twenty-four hours later, cells were infected with SeV (MOI = 0.1) or EMCV (MOI = 1.0) for 12 hours before luciferase assays were performed.

Previously, it has been demonstrated that ASFV infection minimally induces downstream antiviral genes and is highly virulent to pigs [17,22,23]. These observations suggest that ASFV has evolved strategies to antagonize host defenses. Since ASFV AT-rich genome potently induces downstream antiviral genes via RNA Pol-III-RIG-I axis, we investigated whether this axis is targeted by ASFV for immune evasion. To test this, PAMs were pre-infected with ASFV and then transfected with poly(dA:dT). qPCR experiments indicated that poly(dA:dT)-induced transcription of *IFNB1*, *IL6*, and *TNFA* genes was impaired by pre-infection with ASFV (Fig 3A), suggesting that ASFV has evolved strategies to antagonize the Pol-III-RIG-I axis.

To identify ASFV proteins that inhibit the RNA Pol-III-RIG-I axis, we constructed expression plasmids for 145 ORFs of the highly virulent ASFV CN/GS/2018 strain [24], and examined their abilities to inhibit activation of the IFN-β promoter induced by transfected poly(dA:dT) in HEK293T cells, which are deficient in cGAS-mediated signaling [10]. In these screens, we identified ASFV I267L as the most potent inhibitor of poly(dA:dT)-induced activation of the IFN-β promoter in HEK293T cells (Fig 3B). Ectopic expression of I267L impaired activation of the IFN-β promoter induced by transfected poly(dA:dT), ASFV/DNA-AT and poly(I:C) in HEK293T cells (Fig 3C). Similarly, ectopic expression of I267L inhibited *IFNB1* gene transcription induced by transfected poly(dA:dT), ASFV/DNA-AT and poly(I:C) but not ASFV/DNA-GC in porcine PK-15 or human monocytic THP-1 cells (Fig 3D). Interestingly, I267L inhibited transcription of *IFNB1* gene induced by infection of SeV, whose RNA is sensed by RIG-I [25], but not by HSV-1, whose DNA is mostly sensed by cGAS [26] (Fig 3E). These results suggest that I267L inhibits RIG-I- but not cGAS-mediated innate immune responses. Sequence alignment suggests that I267L is a conserved protein between ASFV genotype II and genotype I with amino acid identity of 98.7%. I267L derived from both ASFV genotypes impaired activation of the IFN-β promoter induced by transfected poly(dA:dT), ASFV/DNA-AT, and poly(I:C) in HEK293T cells (Fig 3F), suggesting that the function of I267L in antagonizing RIG-I-mediated innate immune responses is conserved among different genotypes of ASFV.

To determine at which level I267L inhibits RNA Pol-III-RIG-I axis, we examined the effects of I267L on activation of the IFN-β promoter, ISRE and NF-κB mediated by the essential molecules of the RIG-I-like receptors signaling pathways. The results showed that I267L only inhibited signaling induced by RIG-I but not its downstream components including VISA (also called MAVS, Cardif and IPS-1) [27–30], TBK1, TRAF6 or IKKβ [8] (Fig 3G). Interestingly, I267L had no obvious effects on signaling triggered by cGAS-MITA/STING or by MDA5, another viral RNA sensor of the RIG-I-like receptor family [31]. I267L also had no marked effects on RIG-I-CARD, a constitutive active RIG-I truncation mutant [32] (Fig 3G), suggesting that I267L inhibits an event that leads to RIG-I activation. Consistent with these observations, I267L inhibited activation of the IFN-β promoter, ISRE, and NF-κB induced by SeV, whose RNA is mostly sensed by RIG-I, but had no obvious effects on antiviral signaling induced by encephalomyocarditis virus (EMCV), whose RNA is mostly sensed by MDA5 [25] (Fig 3H). These results suggest that ASFV I267L impairs innate immune responses by inhibiting RIG-I activation.

**Fig 4:**
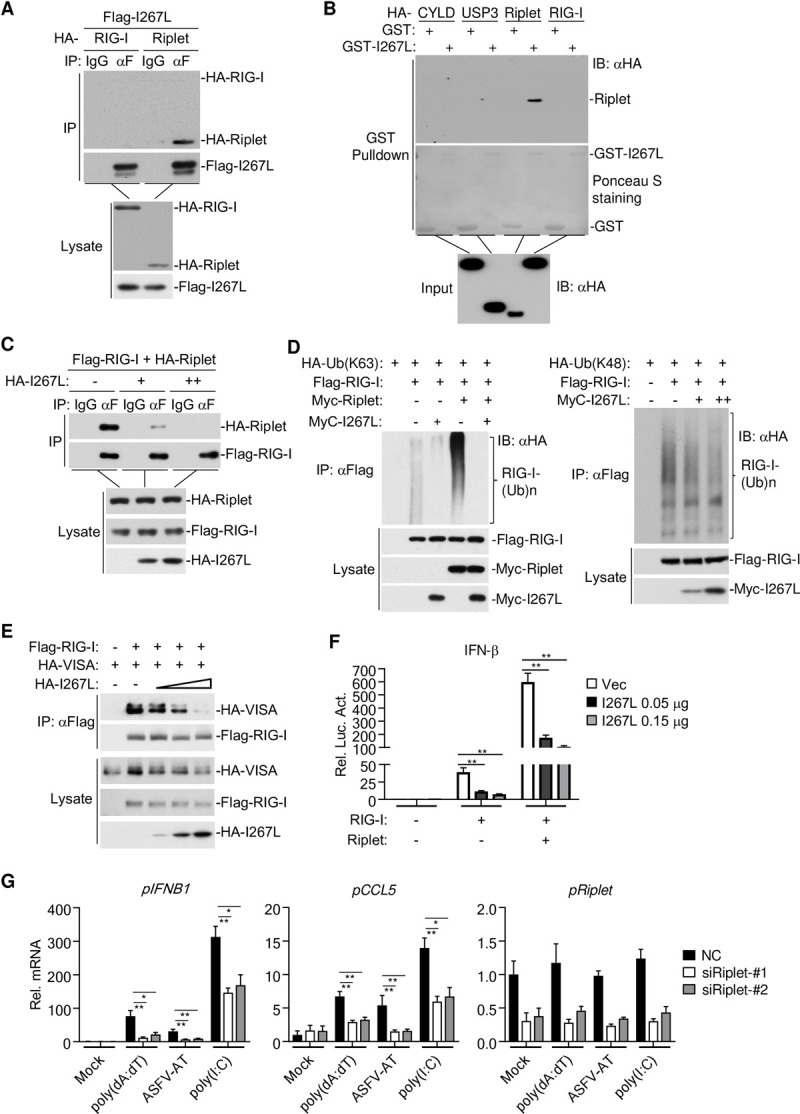
ASFV I267L targets Riplet. (**A**) HEK293T cells (5×10^6^) were co-transfected with Flag-tagged I267L (5 μg) and HA-tagged RIG-I (5 μg) or HA-tagged Riplet (5 μg). Coimmunoprecipitation and immunoblotting were performed with the indicated antibodies. (**B**) HEK293T cells (5×10^6^) were transfected with the indicated plasmids (5 μg). Twenty-four hours later, cell lysates were subjected to pull-down assay with in vitro purified GST or GST-I267L. (**C**) HEK293T cells (5×10^6^) were co-transfected with plasmids of Flag-RIG-I and HA-Riplet (5 μg), and increasing dose plasmids of HA-I267L (0, 1.0, 5.0 μg). Coimmunoprecipitation and immunoblotting were performed with the indicated antibodies. (**D**) HEK293T cells (5×10^6^) were transfected with plasmids as indicated. Twenty-four hours later, coimmunoprecipitation and immunoblotting were performed with the indicated antibodies. (**E**) HEK293T cells (5×10^6^) were transfected with plasmids of Flag-RIG-I and HA-VISA, and increasing dose plasmids of HA-I267L. Coimmunoprecipitation and immunoblotting were performed with the indicated antibodies. (**F**) HEK293T cells (1×10^5^) were co-transfected with the IFN-β promoter reporter, expression plasmids and empty vectors or I267L expression plasmids (0.05 μg) as indicated. (**G**) PK-15 cells (1×10^5^) were transfected with control (NC) or Riplet-siRNA (50 nM). Forty-eight hours later, cells were stimulated with transfected poly(dA:dT), ASFV-AT, and poly(I:C) (1 μg/ml) for 6 hours before RT-qPCR were performed.

We next investigated whether I267L interacts with RIG-I. Mammalian overexpression and coimmunoprecipitation experiments failed to detect an interaction between I267L and RIG-I (Fig 4A). In these experiments, however, I267L interacted with Riplet (Fig 4A), an E3 ubiquitin ligase that catalyzes K63-linked polyubiquitination of RIG-I and which is required for RIG-I activation [33,34]. In vitro pull-down assays showed that I267L interacted with Riplet but not RIG-I or its negative regulators CYLD and USP3 [35–37] (Fig 4B). Overexpression of I267L impaired the interaction between RIG-I and Riplet (Fig 4C). Overexpression of I267L also suppressed Riplet-catalyzed K63-linked polyubiquitination of RIG-I, but had minimal effects on its K48-linked polyubiquitination (Fig 4D). K63-linked polyubiquitination of RIG-I induces its interaction with VISA/MAVS, leading to activation of downstream signaling events [34]. As expected, the recruitment of VISA to RIG-I was impaired in the presence of I267L (Fig 4E). In reporter assays, Riplet robustly potentiated RIG-I-mediated activation of the IFN-β promoter, which was inhibited by I267L (Fig 4F). Knockdown of Riplet in PK-15 cells impaired transcription of *IFNB1* and *CCL5* genes induced by transfected poly(dA:dT), ASFV/DNA-AT and poly(I:C) (Fig 4G). Collectively, these results suggest that I267L inhibits the RNA Pol-III-RIG-I axis by disrupting Riplet-mediated K63-linked polyubiquitination and activation of RIG-I.

### Deficiency of ASFV I267L attenuates its virulence and pathogenesis

**Fig 5:**
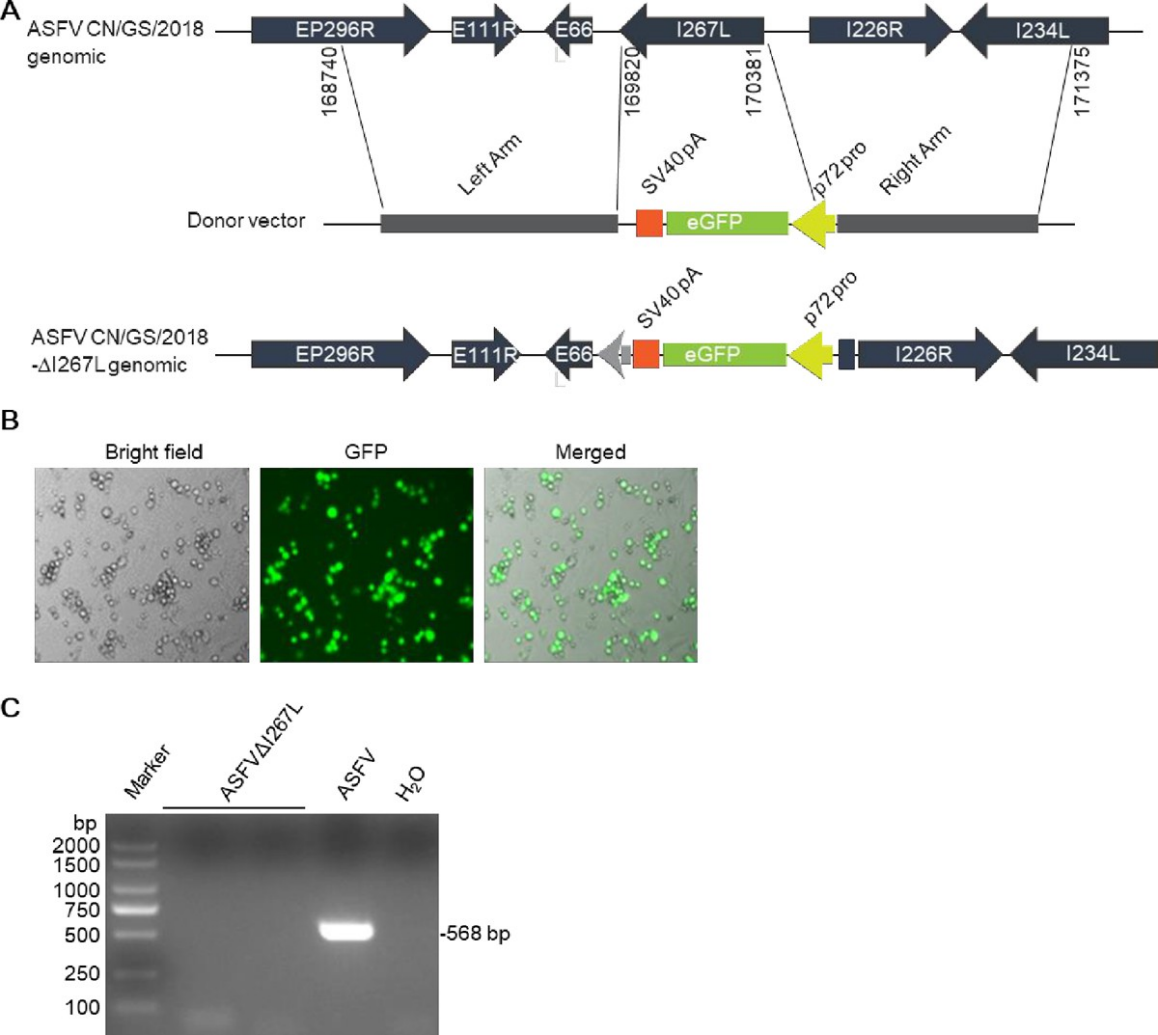
Generation of I267L-deficient virus. (**A**) Schematic presentation of generation of I267L-deletion virus by homologous recombination. (**B**) Confirmation of the successfully recombination by fluorescence microscopy. (**C**) Absence of parental ASFV CN/GS/2018 in ASFVΔI267L virus stock was confirmed by PCR.

To investigate the functions of I267L during ASFV infection, we generated I267L-deficient ASFV (designated as ASFVΔI267L) by genetic modification of the highly virulent ASFV CN/GS/2018 strain using homologous recombination methods. The *I267L* gene was replaced by a cassette encoding the fluorescent eGFP under the control of ASFV p72 promoter (Fig 5A). The mutant virus was purified via 11 rounds of limited dilutions by selecting eGFP-positive clones (Fig 5B). PCR analysis showed that the ASFVΔI267L virus stock was free from parental virus contamination (Fig 5C). To further evaluate the accuracy of the genetic modification and the purity of the recombinant virus stock, full-genome sequences of ASFVΔI267L and parental ASFV were obtained using next-generation sequencing for comparison. The results revealed a deletion of 560 nucleotides (nucleotide 169821 to 170380) from the I267L gene and an insertion of 1191 nucleotides corresponding to the p72-eGFP cassette sequence at this site (S1 Fig). Beside the designed changes, two single nucleotide variations (SNVs) were detected, including one synonymous mutation in CP2475L and one in non-coding region (S1 Fig).

**Fig 6:**
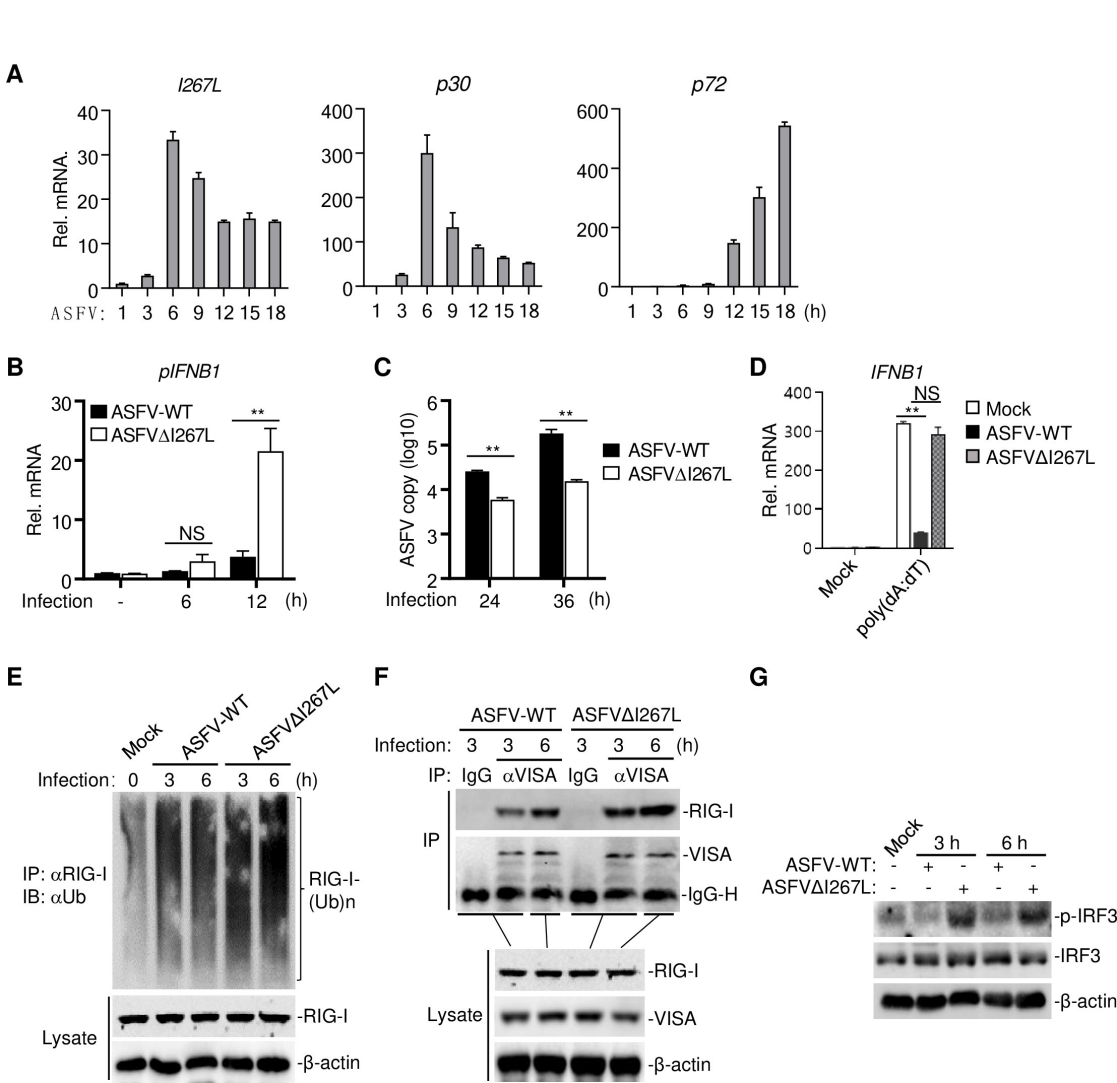
Deficiency of I267L attenuates the ability of ASFV to antagonize innate immune responses. (**A**) Determining the expression level of I267L during ASFV infection by RT-qPCR. The *p30* and *p72* are shown as indicators of early gene and late gene respectively. (**B**) PAMs (1×10^6^) were infected by ASFV-WT or ASFVΔI267L (MOI = 0.1) for 6 hours or 12 hours before RT-qPCR were performed. (**C**) PAMs (1×10^6^) were infected by ASFV-WT or ASFVΔI267L (MOI = 0.1) for 24 hours or 36 hours. Virus DNA was extracted from the cells to determine the viral DNA copies of p72 by qPCR. (**D**) PAMs (2×10^6^) were uninfected or infected with ASFV-WT or ASFVΔI267L (MOI = 0.1) for 6 hours, and then were stimulated with transfected poly(dA:dT) (1 μg/ml) for 4 hours before RT-qPCR were performed. **(E**) PAMs (2×10^7^) were uninfected or infected with ASFV-WT or ASFVΔI267L (MOI = 0.1) for the indicated times. Immunoprecipitation and immunoblotting were performed with the indicated antibodies (2 μg anti-RIG-I antibody was used for each immunoprecipitation). (**F**) PAMs (2x10^7^) were uninfected or infected with ASFV-WT or ASFVΔI267L (MOI = 0.1) for the indicated times. Immunoprecipitation and immunoblotting were performed with the indicated antibodies (2 μg IgG control or anti-RIG-I antibody was used for each immunoprecipitation). IgG-H indicates the heavy chain of IgG. (**G**) PAMs (5×10^6^) were uninfected or infected with ASFV-WT or ASFVΔI267L (MOI = 0.1) for the indicated times. Cells were lysed and subjected to Western blot analysis for examination of the phosphorylation and expression of IRF3. The β-actin were used as loading control.

qPCR analysis indicated that I267L was an early gene whose transcription peaked at 6 hours post infection (Fig 6A), which is consistent with the previous report [38]. We next examined abilities of the parental virus and ASFVΔI267L to induce transcription of the *IFNB1* gene. The results indicated that infection of ASFVΔI267L induced markedly higher mRNA levels of *IFNB1* gene than wild-type ASFV in PAMs (Fig 6B). Consistently, replication of ASFVΔI267L virus in PAMs was markedly suppressed in comparison with wild-type ASFV (Fig 6C). qPCR results showed that deficiency of I267L attenuated the ability of ASFV to antagonize poly(dA:dT)-induced transcription of *IFNB1* (Fig 6D). These results suggest that ASFV I267L inhibits innate immune responses in PAMs. Furthermore, compared with wild-type ASFV, infection of ASFVΔI267L increased RIG-I ubiquitination (Fig 6E), enhanced RIG-I-VISA interaction (Fig 6F), and induced higher levels of IRF3 phosphorylation (Fig 6G), suggesting that deletion of I267L attenuates the ability of ASFV to antagonize RIG-I-mediated signaling.

**Fig 7:**
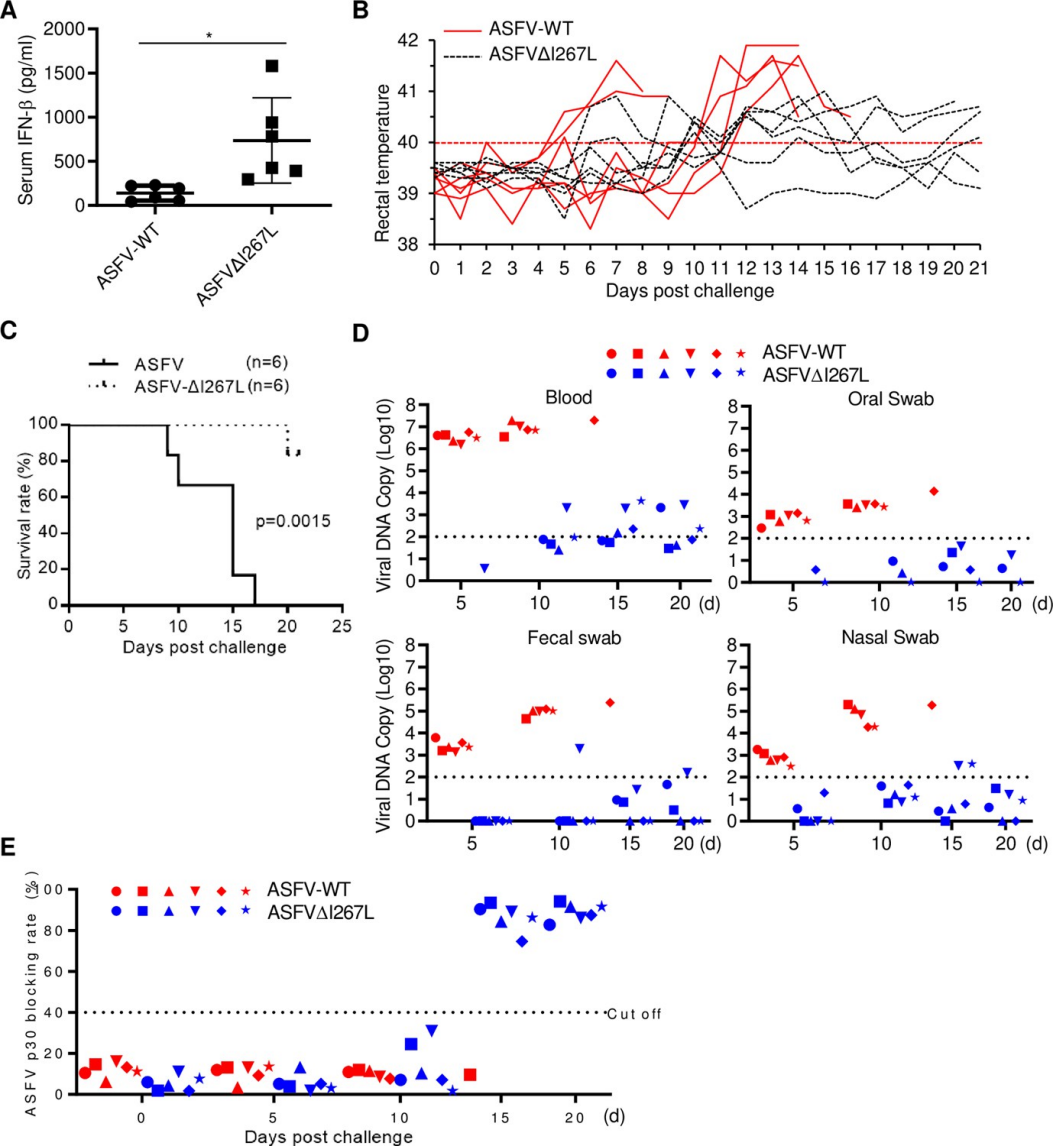
Deficiency of I267L attenuates virulence and pathogenesis of ASFV. (**A**, **B**, **C**, **D** and **E**) Pigs were inoculated intramuscularly (i.m.) with either 10 HAD_50_ of ASFVΔI267L (n = 6) or ASFV-WT (n = 6). The pigs were monitored daily for 21 days for temperature and mortality. The expression level of IFN-β in serum at 5 days post challenge were determined by ELISA (A), the daily temperature changes of pigs were shown in panel (B), the survival rates were shown in panel (C), viral DNA copies in blood, oral swab, fecal swab, and nasal swab collected at 5, 10, 15, and 20 days post challenge were determined by qPCR and shown in panel (D), and the antibodies angaist p30 were determined using an in-house blocking ELISA (E).

To investigate the functions of I267L on virulence and pathogenesis of ASFV, pigs were inoculated intramuscularly with 10 HAD_50_ of wild-type ASFV or ASFVΔI267L virus. Pigs infected with ASFVΔI267L virus produced higher levels of serum IFN-β than those infected with wild-type ASFV at 5 days post-infection (Fig 7A). All six pigs inoculated with wild-type ASFV developed signs of fever with maximum temperature up to 41.6°C (Fig 7B). These pigs also displayed other ASF-related clinical signs such as anorexia, depression, purple skin discoloration, staggering gait and diarrhea. Signs of the disease aggravated progressively over time and all animals died from 9 to 17 days post-infection of wild-type ASFV (Fig 7C). Five out of six pigs inoculated with the ASFVΔI267L virus exhibited mild clinical signs, such as moderate body temperature increase, a short period of anorexia and depression, and survived during the 21-day observation period. One pig inoculated with the ASFVΔI267L virus exhibited partial ASF-related clinical signs and was bedridden from day 16 and died 20 days post-infection (Fig 7B and 7C). In addition, all pigs infected with the ASFVΔI267L virus showed much lower viral loads in blood, oral swab, fecal swab, and nasal swab in comparison with pigs infected with wild-type ASFV (Fig 7D). Previous studies indicate that the levels of circulating antibodies, such as the antibody against p30, are important parameters consistently associated with humoral immune responses [24]. We found that the survival pigs infected with the ASFVΔI267L virus exhibited increased levels of p30 antibody at the late stage of infection (Fig 7E), indicating that the attenuated ASFVΔI267L virus induced better antibody responses. These results suggest that I267L plays an important role in evasion of antiviral innate immune responses and contributes to virulence and pathogenesis of ASFV.

## Discussion

The innate immune response is the first line of host defense against invading pathogens. Recognition of invading viruses by the host PRRs initiates signal cascades to induce type I IFNs and other cytokines, which exhibit antiviral activities through their abilities to induce ISGs and activate the adaptive immune responses. The roles of host PRRs in sensing ASFV have been largely unknown. Recently, it has been reported that attenuated ASFV NH/P68 strain, but not the virulent Armenia/07 strain, activates the cGAS-mediated innate immune signaling at very early phase of infection, indicating that cGAS is functional in sensing ASFV infection and is inhibited by virulent ASFV strains [17]. Whether other DNA sensors are involved in detection of ASFV infection is unknown. The observation that the ASFV genome contains highly AT-rich regions prompted us to evaluate the roles of RNA Pol-III in innate immune sensing of ASFV. Previous studies have demonstrated that RNA Pol-III is involved in sensing AT-rich genome of VZV and innate antiviral responses to VZV [18]. In our study, several lines of evidence suggest that AT-rich genome of ASFV induces innate antiviral responses via the RNA Pol-III-RIG-I signaling pathways. Firstly, knockdown of either cGAS or RIG-I inhibited transcription of *IFNB1* gene induced by ASFV, while simultaneous knockdown of both had collaborative inhibitory effects, suggesting that both cGAS and RNA Pol-III-RIG-I pathways can sense ASFV infection. Second, inhibition of RNA Pol-III by ML-60218 impaired transcription of *IFNB1* gene induced by ASFV. Third, the ASFV genome contains highly AT-rich regions, and synthetic ASFV AT-rich but not GC-rich DNA fragments induced innate immune responses in RIG-I-dependent but cGAS-independent manner. Fourth, RNAs transcribed from ASFV AT-rich DNA fragments or from ASFV-infected cells induced innate immune responses. In addition, RIG-I bound to RNAs transcribed from ASFV AT-rich DNA. These results suggest that the AT-rich DNA of ASFV induces innate immune responses via the RNA Pol-III-RIG-I pathways.

Previous studies have demonstrated that virulent ASFV strains only minimally induce type I IFNs and downstream antiviral genes [6,17]. The simplest explanation for these observations is that ASFV evolves strategies to antagonize innate immune responses. By screening 145 proteins encoded by ASFV, we identified I267L as a potent inhibitor of the RNA Pol-III-RIG-I axis. I267L is a highly conserved protein, which has 100% identity in amino acid sequence among strains of the p72 genotype II, and 100% identity among strains of genotype I ASFV. Our experiments indicated that I267L proteins originated from both genotype II and I could potently inhibit RNA Pol-III-RIG-I signaling, suggesting a conserved strategy of immune evasion by genotype I and II ASFV. It is worth noting that I267L is one of the early genes, which supports a potential role of I267L in evasion of host innate immune responses and contribution to the virulence of ASFV.

Our experiments suggest that I267L inhibits innate immune responses by impairing Riplet-mediated activation of RIG-I. Overexpression of I267L inhibited RIG-I- but not MDA5-, VISA/MAVS-, TBK1- or cGAS-mediated innate immune signaling. However, overexpression of I267L did not inhibit signaling triggered by a constitutive active RIG-I mutant, suggesting that I267L may inhibit an event that leads to RIG-I activation. Previously, it has been shown that K63-linked polyubiquitination of RIG-I catalyzed by the E3 ubiquitin ligase Riplet is essential for RIG-I activation. Our experiments indicated that I267L interacted with Riplet but not RIG-I, disrupted the interaction between Riplet and RIG-I, and impaired Riplet-mediated K63-linked polyubiquitination of RIG-I as well as its recruitment of the downstream adaptor protein VISA. Consistently, I267L inhibited activation of the IFN-β promoter induced by RIG-I and Riplet, whereas knockdown of Riplet inhibited transcription of downstream antiviral genes induced by poly(dA:dT), ASFV/DNA-AT and poly(I:C). These results collectively suggest that I267L abrogates Riplet-mediated RIG-I polyubiquitination and activation.

In our study, we generated I267L-deficient ASFV and examined the roles of I267L in its virulence and pathogenesis. Our results indicated that I267L-deficient virus was dampened in its ability to antagonize RIG-I mediated signaling thus induced higher levels of IFN-β both in PAMs and pigs. Replication of the I267L-deficient virus in PAMs and pigs was compromised in comparison with wild-type ASFV. Importantly, pigs infected with I267L-deficient virus showed less severe clinical signs, such as milder fever, lower viral loads, and higher survival rates comparing with those infected with wild-type ASFV. Furthermore, pigs infected with I267L-deficient virus produced high levels of circulating antibodies. These results suggest that ASFV I267L is an important component for evasion of host innate immune responses and critical determinant of its virulence. Notably, lower virulence of I267L-deficient virus argues RNA Pol-III-RIG-I axis as an important arm of innate sensing to ASFV.

In summary, our findings show for the first time the involvement of RNA Pol-III-RIG-I axis in innate immune responses against ASFV. We also show that ASFV I267L antagonizes the RNA Pol-III-RIG-I axis by impairing Riplet-mediated activation of RIG-I, and therefore, acts as a critical determinant of its virulence. Our study provides important clues for understanding the general landscape of conserved and distinct mechanisms of immune sensing to and evasion by large DNA viruses. Identification of ASFV genes involved in evasion of host immune responses and characterization of underlying mechanisms are recognized as critical steps in the development of vaccines [39]. Identification of I267L as an important inhibitor of innate immune responses and critical determinant of its virulence points to the possibility to develop I267L-deficient attenuated vaccines for ASFV.

## Materials and methods

### Ethics statement

All animals were handled in strict accordance with good animal practices according to the Animal Ethics Procedures and Guidelines of the People’s Republic of China, and the study was approved by the Animal Ethics Committee of Lanzhou Veterinary Research Institute, Chinese Academy of Agricultural Sciences.

### Reagents, antibodies, viruses and cells

Dual-specific luciferase assay kit (Promega), SYBR (Bio-Rad), polybrene (Millipore), poly(I:C) (InvivoGen), RNase A (Thermo Fisher), DNase I (Sigma), mouse antibodies against HA-tag (BioLegend), FLAG-tag (Sigma), Myc-tag (Cell Signaling Technology) and β-actin (Sigma), anti-phospho-IRF3 (Ser396) (Cell Signaling Technology), anti-IRF3 antibody (FL-425) (Santa Cruz Biotechnology), ML-60218 (MCE) were purchased from the indicated manufacturers. Rabbit anti-porcine RIG-I antibody was raised against recombinant porcine C-terminal RIG-I fragment. Mouse monoclonal anti-p30 antibody was raised against a recombinant ASFV CN/GS/2018 p30 protein. SeV, HSV-1 and EMCV were previously described [40,41]. ASFV CN/GS/2018 strain was propagated on PAMs as previously described [17] by the African Swine Fever Regional Laboratory of the Lanzhou Veterinary Research Institute. HEK293T, THP-1 and PK-15 cells were purchased from ATCC. PAMs were prepared by bronchoalveolar lavage as previously described [42]. PAMs were cultured in RPMI 1640 (Gibco) supplemented with 10% fetal bovine serum (Gibco) and 1% penicillin-streptomycin (Thermo Fisher Scientific) at 37°C with 5% CO_2_.

### Constructs

Mammalian expressing plasmids were constructed in pCMV14 or pRK vector by standard molecular biology methods. The point mutation plasmids were constructed by site-directed mutagenesis as previously described [19,27]. Expression plasmids for cGAS, STING/MITA, RIG-I, RIG-I-CARD, MDA5, VISA, TBK1, TRAF6 and IKKβ were previously described [43,44]. Expression plasmids for ASFV ORFs were constructed by insertion of synthetic cDNA for particular ORF into pCMV7.1 vector as described previously [24].

### Synthetic ASFV genomic DNA fragments

AT-rich and GC-rich fragments of the ASFV genome were synthesized and annealed to dsDNA. The sequences of synthesized dsDNAs are as follow:

ASFV/DNA-AT-#1: 5’-ATAAATAACAAGTATATAGGAATATATAGGAATATATA GGAATATATAGAAATATATAGAAATAGCTAAGCTTAATACTAATTCAG-3’; ASFV/DNA-AT-#2: 5’-ATATATAATATAACAAATAATTGTAGCT TAACTATT TTTCCTCATAATGATGTATTTGATACAACATATCAAGTAGTATGGAATCAAATAATTAATTATACAATAAAATTATTA-3’;

ASFV/DNA-GC: 5’-CAGCCGGATGAGCAGGAGCACTCGCGGCCGCAGGTGC GGCCGCCGGCCCGCCAGTTGCCATG-3’.

Excepted for when specifically indicated, ASFV/DNA-AT refers to ASFV/DNA-AT-#1.

### Expression screen assays

HEK293T cells in 48-well plates were transfected with an expression plasmid for ASFV ORF (0.1 μg) and the IFN-β promoter luciferase reporter plasmid (0.05 μg) by standard calcium phosphate precipitation method. To normalize for transfection efficiency, 0.01 μg of pRL-TK (Renilla luciferase) reporter plasmid was added to each transfection. Twenty-four hours later, cells were further transfected with poly(dA:dT) (1 μg/ml) for 10 hours before luciferase assays were performed using a dual-specific luciferase assay kit (Promega) according to the manufacturer’s instructions.

### Coimmunoprecipitation and immunoblotting analysis

Cells were lysed in NP-40 lysis buffer (20 mM Tris-HCl [pH7.4], 150 mM NaCl, 1 mM EDTA, and 1% NP-40) supplemented with protease and phosphatase inhibitors. For each immunoprecipitation, 400 μl of cell lysate was incubated with 0.5 μg of the indicated antibody and 30 μl of 50% slurry of Protein G-Sepharose (GE Healthcare) at 4°C for 2 h. The Sepharose beads were then washed three times with 1 ml of lysis buffer containing 500 mM NaCl. The precipitates were resuspended with 50 μl 2×SDS loading buffer and boiled for 10 min. Immunoblotting analysis was performed following standard procedures.

### RNA extraction and qPCR

Total RNA was extracted using the TRIzol reagent according to the procedures suggested by the manufacturer. cDNA was synthesized using an oligo(dT) primer and M-MLV reverse transcriptase (Invitrogen). qPCR analysis was performed to measure mRNA abundance of the indicated genes. qPCR assays were performed on C1000 Touch Thermal Cycler (Bio-Rad) for 40–45 cycles of thermal denaturation (96°C for 5 s), reannealing and extension (60°C for 30 s). SoAdvanced Universal SYBR Green Supermix (Bio-Rad) were used. Data shown are the relative abundance of the indicated mRNA normalized to that of GAPDH. The sequences of qPCR primer pairs were previously reported [44,45] or are listed below:

porcine *GAPDH*: 5’-ACATGGCCTCCAAGGAGTAAGA-3’,

5’-GATCGAGTTGGGGCTGTGACT-3’;

porcine *IFNB1*: 5’-CACTGGCTGGAATGAAACCG-3’,

5’-AATGGTCATGTCTCCCCTGG-3’;

porcine *IL6*: 5’-CTGCTTCTGGTGATGGCTACTG-3’,

5’-GGCATCACCTTTGGCATCTT-3’;

porcine *TNFA*: 5’-GCCCAAGGACTCAGATCATC-3’,

5’-GGCATTGGCATACCCACTCT-3’;

porcine *CCL5*: 5’-ACACCCTGCTGTTTTTCCTACCT-3’,

5’-AGACGACTGCTGCCATGGA-3’;

porcine *cGAS*: 5’-CCGCCACGAAATCTCAGAAG-3’,

5’-CTCGCTCATAGTAGCTCCCG-3’;

porcine *RIG-I*: 5’-CACGCTTTCGGGGACTATGT-3’,

5’-AAGTGATGCAGCCTCTGTCG-3’;

porcine *Riplet*: 5’-CTGTCAGGAGCCTTCAGAGC-3’,

5’-ACGCCAAGGAAAGGTCTACG-3’;

ASFV *I267L*: 5’- CCCGAGGGTGAATGGATACG-3’,

5’-GGGGCAACAGAGATCGTTCA-3’;

ASFV *p72*: 5’-CCGGGTACAATGGGTCTTCC-3’,

5’-CGCAACGGATATGACTGGGA-3’;

ASFV *p30*: 5’-CTCCGATGAGGGCTCTTGCT-3’,

5’-AGACGGAATCCTCAGCATCTTC-3’.

### RNA Isolation and cell stimulation

To isolate total RNA from PK-15 cells and PAMs, cells (2×10^6^) were lysed in 1 ml TRIzol and total RNAs were extracted according to the procedures suggested by the manufacturer. Extracted RNAs were treated with DNase I (200 U/ml/mg RNA) or RNase A (0.1 mg/ml/mg RNA) at 37°C for 1 hour and then precipitated with isopropanol. The RNAs were then transfected into HEK293T (2 μg/ml) that transfected with IFN-β-luciferase reporter. Six hours later, luciferase assays were performed.

### RNA immunoprecipitation (RIP)

PK-15 cells (2×10^6^) were transfected with ASFV/DNA-AT-#2 (1 μg/ml) for 3 hours. Cells were lysed in RIP buffer (150 mM KCl, 25 mM Tris pH 7.4, 5 mM EDTA, 0.5 mM DTT, 0.5% NP40, 100 U/ml RNase inhibitor, and Protease inhibitor cocktail). The lysate was incubated with control rabbit IgG (2 μg) or an antibody against porcine RIG-I (2 μg) at 4°C for 2 hours. 50 μl protein G beads were washed and added to antibody-cell lysate mixture and incubated at 4°C for 2 hours. The beads were washed 3 times with RIP buffer. The RNA co-precipitated with RIG-I was exacted using the TRIzol reagent. The extracted RNA was treated with DNase I, and reverse transcribed to DNA and analyzed by qPCR with the following pair of primers: 5’-TGTAGCTTAACTATTTTTCCTCA-3’, 5’-TGATTCCATACTACTTGATATGTTG-3’). Proteins associated with the beads were detected by immunoblotting analysis.

### RNA interference

The siRNA duplexes targeting Riplet were chemically synthesized by Gene-Pharma. 25 nM siRNA duplexes were transfected into cells with PepMute siRNA transfection reagent (SignaGen Laboratories) according to the manufacturer’s instructions. After 6 hours, the medium was replaced by fresh medium and cells were further incubated for another 42 hours. The sequences of each siRNA oligonucleotide are as follows:

siRiplet-#1: 5’-AGGAGGAGCAGAAGCTAGA-3’,

siRiplet-#2:5’- CAGGGAAAGTTACAAGAAA-3’;

sicGAS-#1: 5’-GCAGTAACACTTCTGATTA-3’,

sicGAS-#2: 5’-TAATCAGAAGTGTTACTGC-3’;

siRIG-I-#1: 5’-GCAGGCATTTCTGGAGCAA-3’,

siRIG-I-#2: 5’-TTGCTCCAGAAATGCCTGCTT-3’.

### CRISPR-Cas9 knockout

The protocols for genome engineering using the CRISPR/Cas9 system were previously described [44]. Briefly, double-stranded oligonucleotides corresponding to the target sequences were cloned into the lenti-CRISPR-V2 vector, which was co-transfected with packaging plasmids into HEK293T cells. Two days after transfection, the viruses were harvested and used to infect PK-15 cells in the presence of polybrene (8 μg/ml). The infected cells were selected with puromycin (1 μg/ml) for at least 4 days. The following sgRNA sequences were used. porcine RIG-I-#1: 5’-CACTCACCGTCCCTAAACCA-3’; porcine RIG-I-#2: 5’-AAACAACAAGGGCCCGACAG-3’.

### Generation of I267L-deficient virus

The I267L-deficient ASFV were generated by homologous recombination between the parental ASFV CN/GS/2018 genome and recombination transfer vectors by transfection and infection procedures as previously described [24,46]. pUC19 plasmid lacking its multiple cloning sites was used as a backbone, and the recombination cassette was inserted at the SalI and NdeI restriction sites after the T7 promoter as shown in S1A Fig. The left recombination arm lays 1081 bp upstream of I267L which is located at position 168740–169820 in genome of ASFV CN/GS/2018. The right recombination arm lays 995 bp downstream of I267L which is located at position 170381–171375 in genome of ASFV CN/GS/2018. Recombinant transfer vectors were constructed using fusion PCR and the Gibson Assembly technique (Invitrogen Life Sciences, USA). PAMs were transfected with the constructed transfer vectors by using the TransITLT1 transfection reagent (Mirus Bio, Madison, WI, USA) and then infected with ASFV CN/GS/2018 at 24 h post-transfection. The resulting virus from each homologous recombination event was purified by successively picking GFP-positive plaques combined with limited dilution on monolayers of PAMs. The virus obtained from the last round of purification was amplified in PAMs to make a virus stock. Virus DNA was extracted from the virus stock and amplified by PCR to examine whether it was free from parental virus contamination. PCR was performed with the following pair of primers: 5’-AGGGTGAATGGATACGAAGTTTCA-3’ and 5’-ACATGTGTGGTAAACAACATATGG-3’. To further evaluate the accuracy of the genetic modification, full-genome sequences of ASFVΔI267L and parental ASFV were obtained using next-generation sequencing.

### Next-generation sequencing of ASFV genomes

Genomic DNAs of ASFVΔI267L and parental ASFV(CN/GS/2018) were extracted using CTAB/SDS method. Genome sequencing was performed with the Illumina HiSeq platform. The sequencing reads were mapped against the reference sequence through BWA software. SNP, InDel, and SV variations were detected using GATK with the default Parameter, and annotated using SnpEff.

### Virus titration

The wild-type ASFV CN/GS/2018 and I267L-deficient virus (ASFVΔI267L) were quantified by using hemadsorption (HAD) assays as described previously [47] with minor modifications. In brief, PAMs were seeded in 96-well plates. The samples were then added to the plates and titrated in triplicate using 10-fold serial dilutions. HAD was determined at day 7 post-inoculation, and 50% HAD doses (HAD_50_) were calculated by using the Reed and Muench method [48].

### Animal experiments

ASFVΔI267L virus was assessed for its virulence and pathogenesis relative to the parental ASFV CN/GS/2018 virus using 80- to 90-pound commercial breed swine. Twelve pigs were inoculated intramuscularly (i.m.) with either 10 HAD_50_ of ASFV-ΔI267L or ASFV CN/GS/2018 virus. The pigs were monitored daily for 21 days for temperature, clinical signs and mortality. Blood, oral swab, fecal swab, and nasal swab collected at 5, 10, 15, and 20 days post challenge were used for detection of viral DNA, cytokine and p30 antibody levels. Viral loads were determined by qPCR following the OIE-recommended procedure [49]. The target for amplification of the ASFV genome was the conserved p72 gene segment, using the following pair of primers: 5’-CTGCTCATGGTATCAATCTTATCGA-3’ and 5’-GATACCACAAGATC(AG)GCCGT-3’. A TaqMan probe (5’-[6-carboxy-fluorescein (FAM)]-CCACGGGAGGAATACCAACCCAGTG-[6-carboxy-tetramethyl-rhodamine (TAMRA)] -3’) was designed from an alignment of 54 available ASFV sequences at the 3’-end of p72 [50]. The levels of serum IFN-β were determined by ELISA using Interferon-β ELISA kit (Solarbio life sciences, SEKP-0046) according to instructions of the manufacturer. ASFV p30 antibody was measured using an in-house blocking ELISA.

### Biosafety statement and facility

All experiments with live ASFV or its mutant viruses were conducted within the enhanced biosafety level 3 (P3) facilities in the LVRI of the CAAS approved by the Ministry of agriculture and Rural Affairs and China National Accreditation Service for Conformity Assessment.

### Statistical analysis

For two sets of samples, unpaired, two-tailed Student’s t test was used for statistical analysis and the F test was performed to confirm that two populations have the same variances. For multiple comparisons, one-way ANOVA was performed followed by a post hoc test. For the survival curves comparison, log-rank (Mantel-Cox) test was performed. Statistical differences were evaluated using the Graph Pad Prism software.

## Supporting information

S1 Fig Evaluation the accuracy of the genetic modification by next-generation sequencing. (**A**) Sequence analysis of *I267L* gene of ASFVΔI267L. 560 nucleotides (nucleotide 169821 to 170380) of the I267L gene were deleted, and the residual coding sequences of I267L were shown in green font. An insertion of 1191 nucleotides of p72-eGFP cassette was shown in red font. Numbers indicate the nucleotide position of the genome of wild-type ASFV. (**B**) Summary of other mutations of ASFVΔI267L genome. Two single nucleotide variations (SNVs) were detected by next-generation sequencing, including one synonymous mutation and one mutation in non-coding region as listed in the table.
(TIF)
