# Supplementary figures and images for: African swine fever virus I267L acts as an important virulence factor by inhibiting RNA polymerase III-RIG-I-mediated innate immunity
Source: PLoS Pathog. 2022 Jan 28;18(1):e1010270. doi: 10.1371/journal.ppat.1010270 (PMC8827485; doi:10.1371/journal.ppat.1010270)

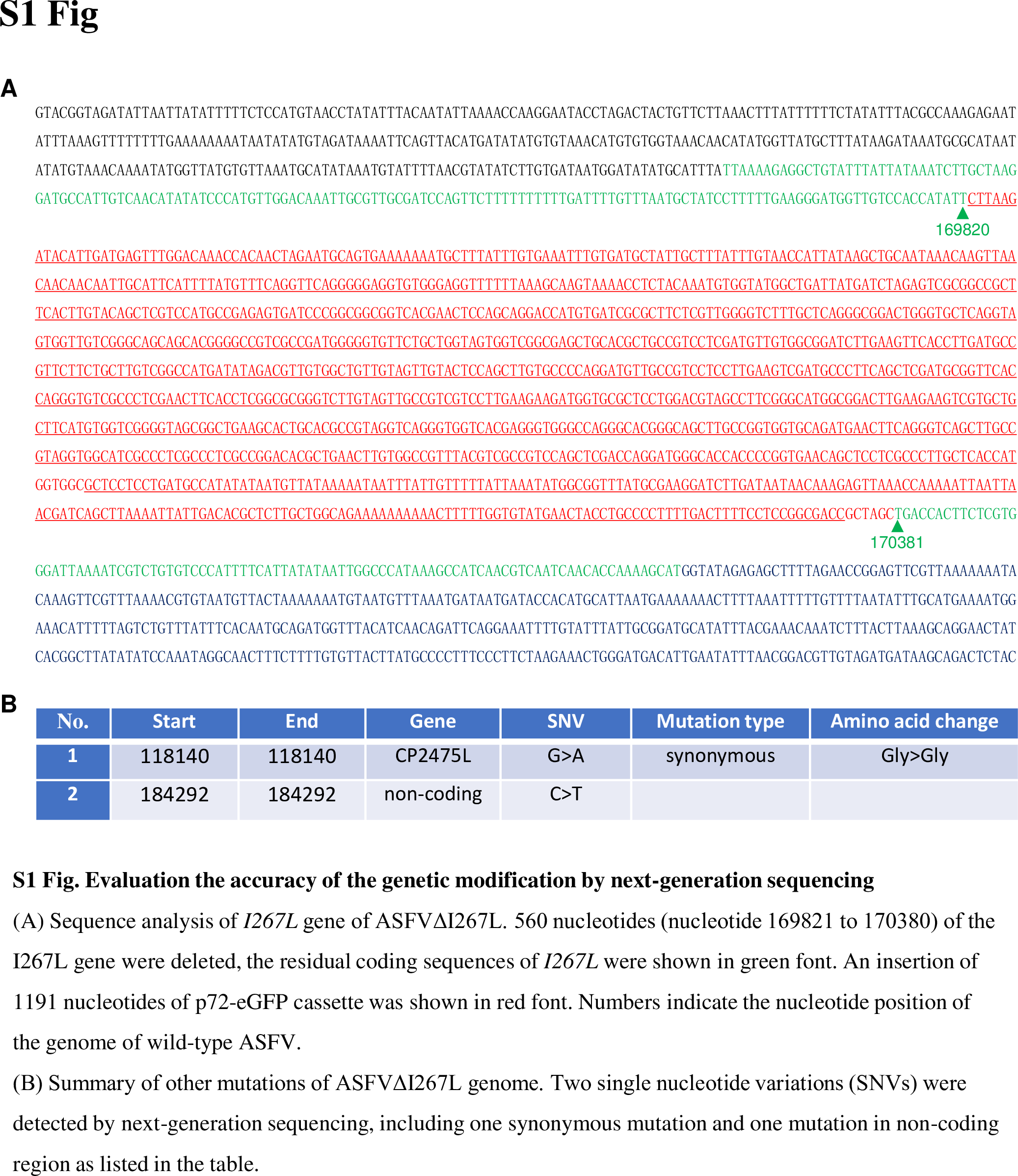

Supplement: S1 Fig — (A) Sequence analysis of I267L gene of ASFVΔI267L. 560 nucleotides (nucleotide 169821 to 170380) of the I267L gene were deleted, and the residual coding sequences of I267L were shown in green font. An insertion of 1191 nucleotides of p72-eGFP cassette was shown in red font. Numbers indicate the nucleotide position of the genome of wild-type ASFV. (B) Summary of other mutations of ASFVΔI267L genome. Two single nucleotide variations (SNVs) were detected by next-generation sequencing, including one synonymous mutation and one mutation in non-coding region as listed in the table. (TIF) [file ppat.1010270.s001.tif]
